# Genomic evolution of *Staphylococcus aureus* isolates colonizing the nares and progressing to bacteremia

**DOI:** 10.1371/journal.pone.0195860

**Published:** 2018-05-03

**Authors:** Jeanne B. Benoit, Daniel N. Frank, Mary T. Bessesen

**Affiliations:** 1 Division of Infectious Diseases, Department of Medicine, University of Colorado Denver, Aurora, Colorado, United States of America; 2 Department of Veterans Affairs Eastern Colorado Healthcare System, Denver, Colorado, United States of America; Universitatsklinikum Hamburg-Eppendorf, GERMANY

## Abstract

**Background:**

Nasal colonization by *Staphylococcus aureus* is a key risk factor for bacteremia. The objective of this study is to identify genomic modifications occurring in nasal carriage strains of *S*. *aureus* as they progress to bacteremia in a cohort of hospitalized patients.

**Methods:**

Eight patients with *S*. *aureus* bacteremia were identified. Genomic sequences of the bloodstream isolates were compared with 57 nasal isolates collected longitudinally prior to the occurrence of bacteremia, which covered a timespan of up to 326 days before bacteremia.

**Results:**

Within each subject, nasal colonizing strains were closely related to bacteremia strains. Within a subject, the number of single nucleotide polymorphisms (SNPs) observed between time points was greater than within a single time point. Co-colonization and strain replacement were observed in one case. In all cases colonization progressed to bacteremia without addition of new virulence genes. In one case, a mutation in the accessory gene regulator gene caused abrogation of *agr* function.

**Conclusion:**

*S*. *aureus* evolves in the human nares at a variable rate. Progression of *S*. *aureus* nasal colonization to nosocomial infection is seldom associated with acquisition of new virulence determinants. Mutation in the *agr* gene with abrogation of function was associated with progression to bacteremia in one case.

## Introduction

*Staphylococcus aureus* bloodstream infections are an important clinical problem, with associated mortality of 7% to 29% [1–3]. Nasal colonization is the antecedent to bloodstream infection in most cases [4]. When nasal colonization is identified prior to bloodstream infection, pulsed field gel electrophoresis analyses show that the nasal and bloodstream isolates match in 80% of cases, confirming that the nasal colonizer is the source of the bloodstream infection [5]. Despite evidence that *S*. *aureus* colonization increases the risk of infection, the majority of colonized individuals remain free of infection. A clear understanding of the reasons that some colonized individuals develop infection, while others remain healthy, would inform efforts to control this important pathogen.

The events that cause colonization to progress to invasive disease are poorly understood. Clinical risk factors for invasive infections with *S*. *aureus* include end stage renal disease, central venous catheterization, and chronic wounds [6]. These risk factors alone do not explain why some colonized people remain free of infection, while others go on to suffer invasive disease. The innate immune system is activated in patients with *S*. *aureus* nasal colonization, but is ineffective in clearing carriage in persistently colonized persons [7]. Humoral immunity provides some protection against mortality when patients become bacteremic, but it does not prevent bacteremia [8].

Focusing on the pathogen component of the host-pathogen relationship, one may hypothesize that changes in *S*. *aureus* virulence during carriage could lead to invasive infection. Recent studies have used whole genome sequencing to capture the population dynamics and evolution of *S*. *aureus* strains that have colonized the nares and resulted in a single case of bacteremia [9] and four cases of recurrent soft tissue infections [10]. To further elucidate the evolutionary strategy of *S*. *aureus* strains colonizing the nares, we performed a genomics-based, cohort study of eight patients who were nasal *S*. *aureus* carriers and subsequently developed *S*. *aureus* bacteremia.

## Materials and methods

### Study design

Samples of MRSA nasal colonizing strains obtained and stored for an active methicillin-resistant *S*. *aureus* (MRSA) screening and contact precautions program, as described previously, were used for the study [11]. All bloodstream isolates of *S*. *aureus*, including MRSA and methicillin sensitive *S*. *aureus* (MSSA), were obtained through standard clinical laboratory procedures and stored at -80°C. Methicillin-sensitive *S*. *aureus* (MSSA) isolates from nasal swabs were retrieved from storage, and cultured (see below). Clinical data were abstracted from the electronic medical record, and used to calculate the Charlson comorbidity index at the time of the bloodstream infection [12].

### Ethics declaration

The study was approved by the Colorado Multiple Institutional Review Board, protocol #12–0346.

### Microbial Culture

We identified all patients with *S*. *aureus* bacteremia dated 7/1/2012–6/30/2014, and retrieved blood culture and nasal isolates from stored glycerol stocks. Frozen archived nasal swabs were thawed on ice, suspended in TE buffer (10mM Tris pH 8.0, 1 mM EDTA) by vortexing, and plated on mannitol salt agar and sheep’s blood agar plates. Petri plates were evaluated after incubation at 37°C (without CO_2_) for 24 hours. Individual isolates were glycerol stocked and stored at -80°C. For the blood isolates, susceptibility to oxacillin was tested on the Vitek platform, according to manufacturers’ instructions. *S*. *aureus* ATCC type strains BAA-1699 (USA100) and BAA-1556 (USA300) were also glycerol stocked and sequenced.

### RNAIII qRT-PCR

Following the method described in Laabei, et al. [13] *agr* functionality was tested by qRT-PCR assay of the RNAIII gene and *gyrB* (housekeeping gene). Overnight cultures were diluted 1:100 in TSB media and cells grown for 8 hours. Cell pellets were collected on ice and flash frozen. RNA was extracted using a Qiagen RNeasy Mini kit with on-column DNAse I digestion. cDNA was generated using SuperScript II Reverse Transcriptase (Invitrogen) according to manufacturer’s directions using random hexamers (Qiagen). The cDNA was then used for qRT-PCR using DyNAmo ColorFlash SYBR Green qPCR kit (ThermoFisher Scientific) following the manufacturer’s recommendations. qRT-PCR was performed on a BIO-RAD CFX96 Touch Real-Time PCR Detection System with the following protocol: 95°C for 7 minutes followed by 40 cycles of 95°C for 15 seconds and 60°C for 60 seconds. For each reaction, the ratio of RNAIII and *gyrB* transcript number was calculated as follows: 2 ^(Ct gyrB-Ct RNAIII)^.

### DNA extraction and genomic sequencing

For genomic DNA sequencing, *S*. *aureus* glycerol stocks were streaked for single colonies on tryptic soy agar plates. Single colonies were grown overnight in 3 mL BHI media. DNA was extracted from cell pellets by mechanical and thermal lysis in a buffer of 10mM Tris, 1mM EDTA, 0.5% NP40 [14, 15]. DNA concentrations were measured using a Qubit 2.0 fluorometer following the manufacturer’s protocol for dsDNA HS Assay Kit (Life Technologies) and DNA diluted to 0.2 ng/μL. The NexteraXT DNA sample preparation kit (Illumina, USA) was used to prepare 1 ng of DNA for sequencing on the Illumina MiSeq platform, following the manufacturer’s protocol. Paired-end sequencing of multiplexed samples (20–24 samples per sequencer run) was performed using the Illumina MiSeq 600 cycle version 3 reagent kit. For comparison, draft genomes of USA100 (BAA-1699) and USA300 (ATCC-1556) reference strains were also generated in parallel.

### Data analysis

To identify SNPs between pairs of isolates, we used Bowtie2 (v2.2.6) software [16] to map paired-end reads to the *S*. *aureus* reference sequence USA300_FPR3757 (ATCC-1556) [17]. The USA300_FPR3757 contains three annotated plasmids (pUSA01, pUSA02, and pUSA03). Duplicates were marked and excluded from further analysis using Picard2 RemoveDuplicates tool (http://broadinstitute.github.io/picard). SNPs were identified with Freebayes v1.0.2.29 [18] using a list (-L) to compare all genomes from a single subject compared to the reference sequence (USA300_FPR3757) and filtered using SnpSift [19] for the following qualities (MQM > 20 and DP > 40). For each subject, the SnpSift call for Reference (isRef) and Variant (isVar) was completed in a stepwise manner to determine how mutations accumulated over the subject’s history. SNPs were annotated from the USA300_FPR3757 genome and classified into functional categories using TIGRFAM [20] and Aureowiki [21].

The A5 *de novo* assembly pipeline [22] was used to construct draft genome assemblies (contigs) from the raw paired-end sequence reads (see GenBank accession numbers PHUU00000000.1- PHWY00000000.1 for published draft genome assemblies). Virulence factors were identified among each set of contigs using the *S*. *aureus* VirulenceFinder version 1.5 database [23]. Multi-locus sequencing type (MLST) assignments were made by searching each set of contigs for sequences of the housekeeping genes (*arcC*, *aroE*, *glpF*, *gmk*, *pta*, *tpi*, *yqiL*) and comparing these to a database of *S*. *aureus* types for MLST [24]. Mykrobe predictor was used to predict the antibiotic resistant profiles from the A5 pipeline results [25].

To infer the evolutionary relationships among the *S*. *aureus* isolates, sequence reads were mapped to a USA300 reference genome (USA300_FPR3757). For each isolate, informative base calls were made only at positions with coverage of >10 reads and >90% concordance in base calls across all aligned reads; positions not meeting these criteria were called as “N” and excluded from subsequent analyses. Read-mapping created a genome-wide multiple sequence alignment (>2.8 million positions) of all included genomic sequences [26], from which an approximately-maximum-likelihood phylogenetic tree was generated using FastTree v2.1.10 [27] and displayed with Dendroscope v3.5.9 [28]. Because no full-length, polished genomic sequences of USA100 were available, we corroborated results by generating trees using as references concatenated contigs USA100 isolate that was sequenced in our laboratory, along with two other full-length reference sequences, NC002953 MSSA (MSSA 476), and NC002951 COL (*Staphylococcus aureus subsp*. *aureus* COL).

## Results

### Study population

**Fig 1:**
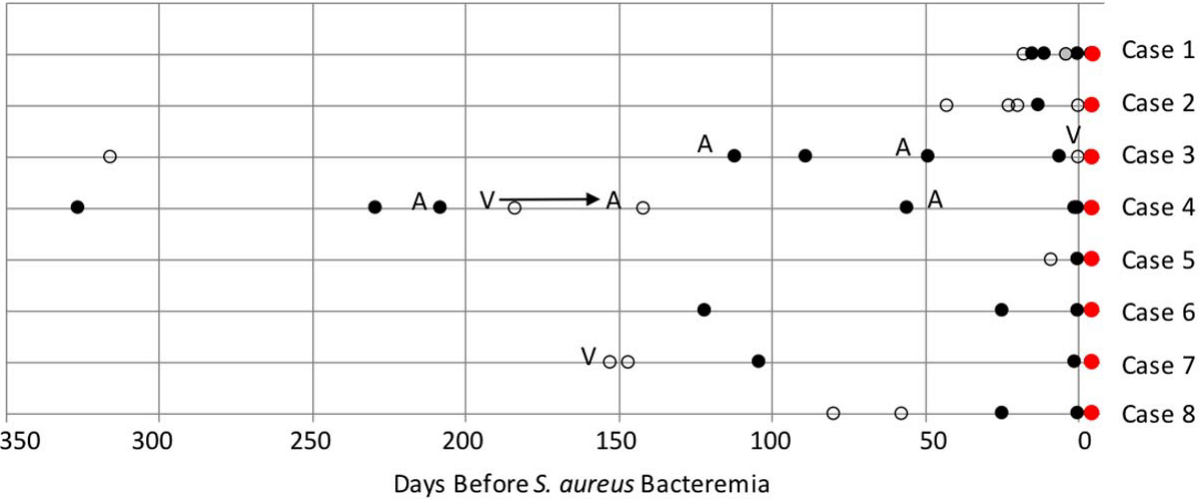
Time line of nares swabs leading to bacteremia. Circles indicate nares swab available, red filled circles indicate a bactermia (blood) isolate sequenced, black filled circles indicate *S*. *aureus* isolate sequenced, unfilled circles indicate no *S*. *aureus* isolate available to be sequenced, grey filled circles indicate that a *S*. *aureus* isolate is available but not sequenced, A represents antibiotic given, and V represents Vancomycin given. (See Table 1 for more information).

During the study period there were 64 cases of S. aureus bacteremia identified by prospective weekly review of Clinical Microbiology records. Serial nasal isolates prior to onset of bacteremia were available for eight cases. Those eight subjects were included in this longitudinal study of *S*. *aureus* isolates collected 1–326 days (63 +/- 108 days, mean +/- SD) prior to onset of bacteremia (Table 1 and Fig 1). The case subjects consisted of mostly men (7/8), median age 64 years (range 62–92), and Charlson comorbidity index [12] of 3.63 +/- 1.87 (mean +/- SD). Review of cases 4 and 7, which both transiently carried an unusual MRSA strain, identified no transmission opportunities, i.e. there were no shared care providers, or locations of care on wards, clinics or in Imaging. Six of eight bacteremia isolates were MRSA (Table 2). Draft genome sequences were generated for 57 isolates, including three individual replicate isolates for the nasal swabs closest to and farthest in time from the blood isolate (See Tables 1 and S1). A single colony from each blood culture was selected for sequencing due to the previous observation by Laabei et al that there was no genetic diversity when they sequenced 12 isolates sampled at two time points and collected in three bottles from patients with *S*. *aureus* bacteremia [29]. For all subjects, the bacteremia isolate is noted as ‘B0’ whereas the isolates collected from the nares are denoted as N, followed by the number of days prior to bacteremia from which the specimen was collected.

**Table 1:** Patient characteristics and sampling time points.

Time line^a^	Clinical Features^b^	Charlson score
Case 1	62 y/o man with heart failure admitted for diuresis	2
T18	Admission to hospital	
T15	Nares culture positive for MSSA (isolates sequenced-N15a, N15b, N15c).	
T11	Nares culture positive for MSSA (isolate sequenced- N11).	
T0	Nares culture positive for MSSA (isolates sequenced- N0a, N0b, N0c). Two of two sets of blood cultures positive for MSSA (isolate sequenced—B0).	
Case 2	65 y/o man with squamous cell cancer of the base of the tongue and pleura.	6
T21	Nares screen negative for MRSA	
T22	Video assisted thoracoscopic surgery and talc pleurodesis.	
T20	Nares screen negative for MRSA	
T13	Nares screen positive for MRSA (isolates sequenced–N13a, N13b, N13c)	
T1	Purulent drainage chest tube site	
T0	Nares screen positive for MRSA. One of two sets of blood cultures positive for MRSA (isolate sequenced–B0)	
Case 3	62 y/o female with diastolic heart failure, chronic obstructive pulmonary disease, obstructive sleep apnea and obesity hypoventilation syndrome admitted at T-0 with respiratory failure.	5
T108	Admitted with exacerbation of chronic obstructive pulmonary disease. Treated with prednisone, beta agonists and azithromycin (isolates sequenced–N108a, N108b, N108c).	
T85	Admitted with atrial fibrillation diastolic dysfunction and fluid overload (isolate sequence–N85).	
T60	Treated with amoxicillin for sinusitis	
T45	Admitted for exacerbation of heart failure (isolate sequenced–N45).	
T2	Admitted for exacerbation of heart failure (isolate sequenced–N2a, N2b, N2c).	
T0	Blood culture positive *S. aureus* (isolate sequenced–B0).	
Case 4	92-year-old man with coronary artery disease and end stage renal disease receiving hemodialysis via a tunneled catheter, admitted with sepsis.	3
T326	Admitted with hypotension due to volume shifts during hemodialysis (isolate sequenced–N326).	
T229	Screening nasal swab obtained (isolate sequenced–N229).	
T212	Cefazolin x 1 for prophylaxis for hemodialysis catheter placement	
T208	Admitted for heart failure and cardiogenic pleural effusion. Screening nasal swab obtained (isolate sequenced–N208).	
T201	Admitted to outside hospital for MRSA bacteremia. Treated with 6 weeks IV vancomycin.	
T175-T160	Vancomycin after each hemodialysis session.	
T142-T56	Admitted to VA Nursing Home for physical rehabilitation	
T56	Discharged after a stay for management of heart failure, screening nasal swab obtained (isolates sequenced–N56a, N56b, N56c).	
T50	Cefazolin x 1 for prophylaxis with HD catheter placement	
T30-T18	Cefazolin after each session of hemodialysis for cellulitis at hemodialysis catheter site.	
T0	Central venous catheter associated bloodstream infection related to hemodialysis catheter (isolates sequenced N0a N0b, N0c, B0).	
Case 5	63 y/o man was admitted for upper gastrointestinal hemorrhage due to gastric cancer. Past history of end stage renal disease due to IgA nephropathy, dialyzed via right internal jugular tunneled catheter. Nasal MRSA screening cultures and PCR had been persistently positive ~1.5 years.	5
T12- T8	Peripherally inserted central catheter (PICC) placed for transfusion support. Multiple blood transfusions for GI bleeding.	
T3	Patient received first of planned 5 fractions of palliative X-ray therapy to control UGI bleeding	
T1	Patient received second dose of XRT.	
T0	He developed fever, with no localizing signs, blood cultures were positive for MRSA (isolates sequenced–N0, B0).	
Case 6	83 y/o man with myelodysplastic syndrome that evolved to acute myelogenous leukemia. He had persistent MRSA nares colonization documented for 17 months prior to first sample on T-122	5
T122	Brief hospital admission for flank pain that was found to be musculoskeletal in origin. Nares screen positive for MRSA (isolates sequenced–N122a, N122b, N122c).	
T25	Patient presented with fever to 102^0^ F, and no localizing findings. Urinalysis was normal; urine culture grew ampicillin sensitive Enterococcus species. Blood cultures were negative. Antimicrobial therapy was not administered and fevers resolved spontaneously. Nares screen positive for MRSA (isolate sequenced–N25).	
T22	Patient received his weekly dose of romiplostim (thrombopoietin analog).	
T14	Patient received his weekly dose of romiplostim (thrombopoietin analog).	
T7—T0	Fever without localizing signs, attributed to urinary tract infection. Patient treated with ciprofloxacin.	
T0	Fever and altered mental status noted. No venous access was in place at the time of presentation. Urine and blood cultures grew MRSA (isolates sequenced–N0a, N0b, N0c, B0).	
Case 7	85 y/o man with a history of a left renal laceration from ski accident 12 years previously Multiple negative nares screening tests for MRSA over 2 years prior to first positive screening test.	0
T153	Renal abscess was drained percutaneously and yielded 580 cc pus which grew large number of pansensitive coagulase negative staphylococci, not Staphylococcus lugdunensis. He was treated with vancomycin plus pipracillin-tazobactam for 4 days, then with cotrimoxazole for 30 days.	
T104	Nares screening test positive for MRSA (isolates sequenced–N104a, N104b, N104c).	
T1	Nares screening test positive for MRSA (isolates sequenced–N1a, N1b, N1c).	
T0	Renal abscess aspirated, culture grew MRSA. One of two blood cultures grew MRSA (isolate sequenced–B0).	
Case 8	62 year old man with peripheral arterial disease and lower extremity ulcers.	3
T80	Initial visit for evaluation of peripheral arterial disease, and follow-up of revascularizaton surgery at outside hospital. Nares Cepheid PCR screening test positive for MRSA (isolate sequenced, *Staphylococcus haemolyticus*).	
T58	Admitted with gangrene right foot due to peripheral arterial insufficiency. Nares Cepheid PCR screening test positive for MRSA. (no isolate sequenced, 0 colonies on blood agar or mannitol salt)	
T36	Right below knee amputation for uncontrolled infection of foot and peripheral arterial disease.	
T25	Nares screening test positive for MRSA (isolates sequenced–N25a, N25b, N25c).	
T0	Admitted to hospital with sepsis syndrome. Two of two sets of blood cultures positive for MRSA (isolates sequenced–N0a, N0b, N0c, B0).	

^a^Time zero, T0, is the date of the positive blood culture.

^b^Nasal organisms are identified with the number of days prior to the positive blood culture, e.g. N15 was isolated from a nasal swab obtained 15 days prior to the positive blood culture (B0).

**Table 2:** Overview of blood isolate strain types inferred *in silico* from genomic sequencing results.

	MLST	Methicillin Resistance	SCC Mec
Case 1	8	MSSA	NA
Case 2	8	MRSA	IV
Case 3	5	MSSA	NA
Case 4	5	MRSA	II
Case 5	5	MRSA	II
Case 6	5	MRSA	II
Case 7	5	MRSA	II
Case 8	5	MRSA	II

### Sequence analyses

**Fig 2:**
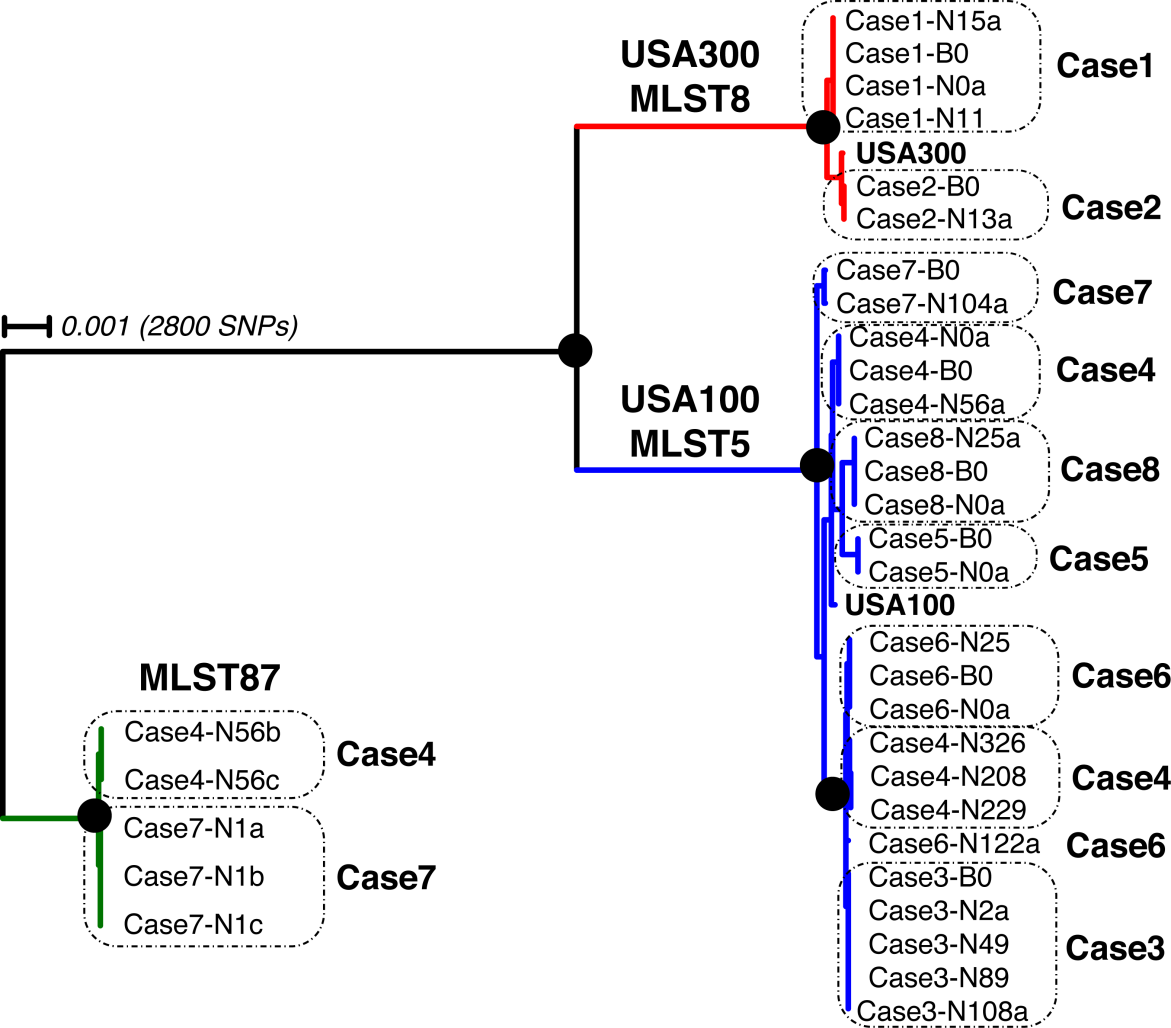
Phylogenetic relationships between *S. aureus* nasal and blood isolates. Whole-genome multiple sequence alignments were generated and a maximum-likelihood phylogenetic tree calculated using Fasttree v2.1.10 (40), as described in the text. Multi-locus sequence types were inferred from assembled genomic sequences. A selection of nodes with bootstrap scores >99% are marked with closed circles (all bootstrap scores are indicated in the cladogram in S1 Fig). The scale bar denotes 0.001 base substitutions per position, which is equivalent to ~2800 SNPs over the 2,800,000 genomic positions”.

**Fig 3:**
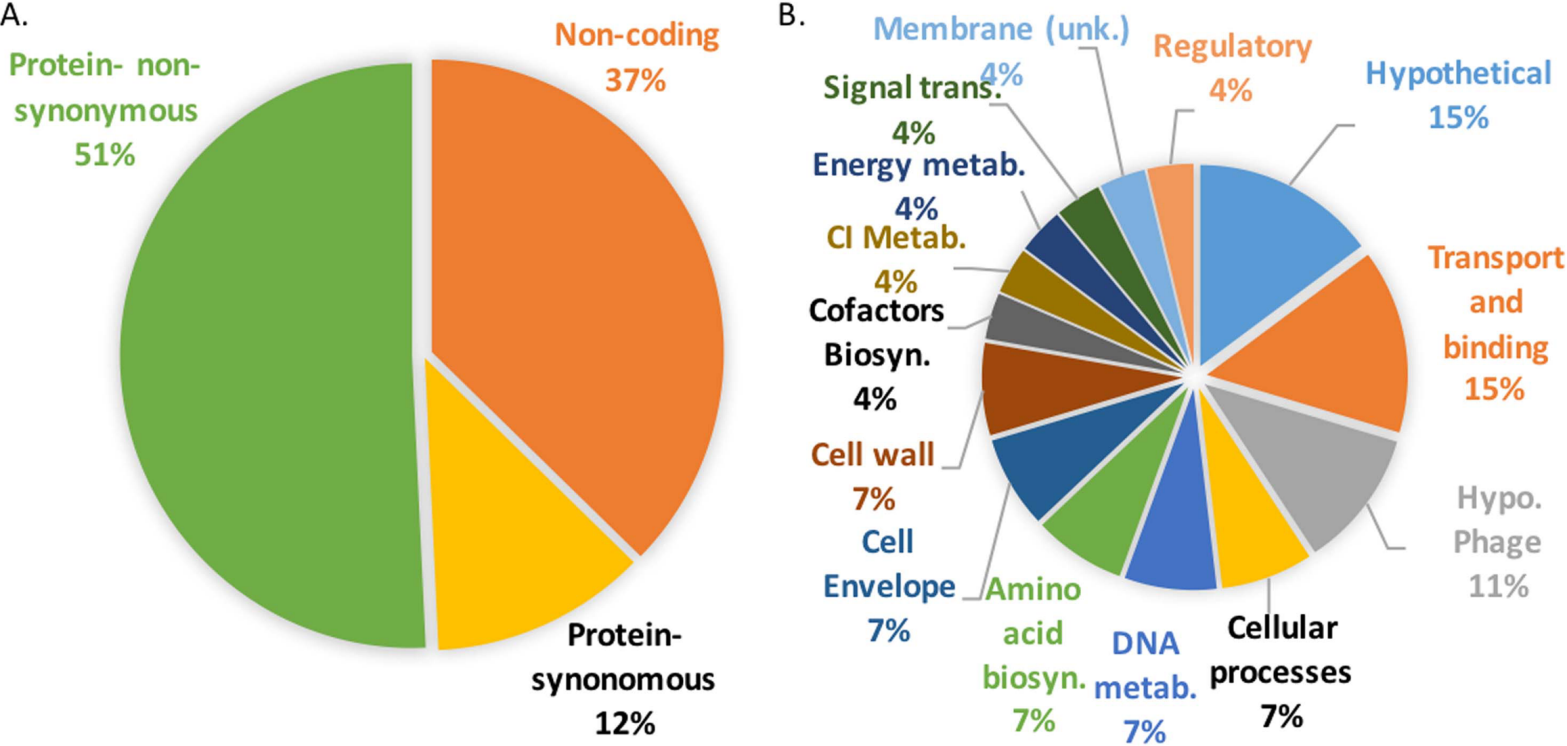
Distribution of SNPs. A) Distribution of all SNPs identified in *S. aureus* isolates. B) Distribution of Non-Synonymous SNPs identified in *S. aureus* isolates by functional categories defined by Aureowiki.

By mapping reads from each draft genome to a USA300 reference genome sequence (USA300_FPR3757, ATCC-1556), we created a whole-genome multiple-sequence alignment of 2.8 million base pairs. Dendrograms inferred from these alignments (Figs 2 and S1) show that isolates from two subjects clustered closely with the USA300 reference sequence (<700 SNPs), while most of the remaining genomes clustered near each other and with the USA100 reference sequence (<1600 SNPs). In comparison, >23,000 SNPs were detected through pairwise comparisons of members of the USA300 and USA100 clusters (Fig 3). For instance, pairwise comparison of USA100 and USA300 type strains comprised 2,655,116 informative positions (i.e., coverage >10 bases for each sequence, with >90% of reads concordant for the base call at that position), with base differences identified at 22,339 (0.84%) positions. In general, all isolates obtained from an individual clustered closely to one another; however, some exceptions were noted and will be discussed below (see Case 4, Case 6, and Case 7; S2 Table). Through the *in silico* MLST analysis, all of the strains clustering with USA300 and USA100 reference strains were assigned to MLST 8 and MLST 5, respectively. Five additional isolates (from two time points) clustered together and were assigned to MLST 87 (discussed below in Case 4 and Case 7).

Whole-genome multiple sequence alignments were generated and a maximum-likelihood phylogenetic tree calculated using Fasttree, as described in the text. Multi-locus sequence types were inferred from assembled genomic sequences. Multi-locus sequence types were inferred from assembled genomic sequences. A selection of nodes with bootstrap scores >99% are marked with closed circles (all bootstrap scores are indicated in the cladogram in S1 Fig). The scale bar denotes 0.001 base substitutions per position, which is equivalent to ~2800 SNPs over the 2,800,000 genomic positions analyzed.

Next, VirulenceFinder software was used to infer the virulence gene profile of isolates from each subject (Table 3). For simplicity, we restricted this analysis to comparing the earliest nasal isolate with the corresponding B0 isolate from each subject. In all cases, the virulence profile (presence or absence of each gene) of the nasal isolate was identical to that of its paired B0 isolate. The MLST 8 strains encoded a smaller number of virulence genes compared with the MLST 5 strains, while the MLST 87 strains differed notably from both the MLST 5 and MLST 8 strains (Table 3). Antibiotic susceptibility profiles of each B0 isolate and the earliest nasal isolate from each subject were inferred *in silico* and compared using Mykrobe predictor analysis [25]. No differences were observed in the predicted antibiotic susceptibility profiles between pairs of isolates from each subject (data not shown). Comparison of the *in silico* antibiotic susceptibility profiles to those determined using the Vitek platform showed concordant results in most cases (S3 Table). In the sole exception, Case 7, the prediction of ciprofloxacin sensitivity did not match the Vitek results, which indicated resistance. However, ciprofloxacin has been shown to have a higher false negative prediction rate compared to other antibiotics [25].

**Table 3:** Virulence determinants identified in *S*. *aureus* isolate genomes.

Case number	1	2	3	4	5	6	7	8	7
Specimen source	B	B	B	B	B	B	B	B	N1
MLST Type	8	8	5	5	5	5	5	5	87
*aur* (aureolysin)	P	P	P	P	P	P	P	P	P
*hlb* (β-hemolysin)	P	P	P	P	P	P	P	P	P
*hlgA* (δ-hemolysin component A)	P	P	P	P	P	P	P	P	P
*hlgB* (δ-hemolysin component B)	P	P	P	P	P	P	P	P	P
*hlgC* (δ-hemolysin component C)	P	P	P	P	P	P	P	P	P
*lukD* (leukocidin E)	P	P	P	P	P	P	P	P	
*lukE* (leukocidin M)	P	P	P	P	P	P	P	P	
*sak* (staphylokinase)	P	P	P	P	P	P	P	P	P
*scn* (Staph complement inhibitor)	P	P	P	P	P	P	P	P	P
*splA* (serine protease-like A)	P	P	P	P	P	P	P	P	
*splB* (serine protease-like B)	P	P	P	P	P	P	P	P	
*seb* (enterotoxin B)									P
*sed* (enterotoxin D)				P	P	P			
*seg* (enterotoxin G)				P	P	P	P	P	
*sei* (enterotoxin I)			P	P	P	P	P	P	
*sej* (enterotoxin J)			P	P	P	P			
*sek* (enterotoxin K)									P
*sem* (enterotoxin M)			P	P	P	P	P	P	
*sen* (enterotoxin N)			P	P	P	P	P	P	
*seo* (enterotoxin O)			P	P	P	P	P	P	
*seq* (enterotoxin Q)									P
*ser* (enterotoxin R)				P	P	P			
*seu* (enterotoxin U)				P	P	P	P	P	

B, blood; N1, nares at time 1; P, present.

SNPs were identified from consensus draft genomes generated through Bowtie2 read-mapping to a USA300 reference genome (USA300_FPR3757) [17]. Each genomic sequence was compared within a subject using Freebayes and SnpSift to identify SNPs common within the set of genomic sequences. Forty-two percent of the SNPs resulted in a predicted amino acid change within a protein coding region (Table 4 and Fig 3), 29% were detected in intergenic regions (Tables 5 and S4), and 12% resulted in synonymous mutations within a protein coding region (S5 Table). To identify the functions of genes harboring non-synonymous mutations (Fig 3B), we queried the Aureowiki [21] which utilizes the TIGRFAM classification system [20]. Hypothetical proteins (18%), and transport and binding proteins (18%), were the two most highly represented functional classes, although a great diversity of functions were observed across the entire set of SNPs (Table 4 and Fig 3B).

**Table 4:** Accumulation of non-synonymous single nucleotiode polymorphisms.

CASE	USA300POS.	ISREF / ISVAR	GENE	CODON	AA CHANGE	GENE NAME/FUNCTION	TIGRFAM CATEGORY	ASSOCIATION
**CASE 1**	2063390	N15 / N11, N0, B0	*sdcS*	gCc/gAc	Gly—Asp	Sodium-dependent dicarboxylate transporter	Transport and binding	-
**CASE 2**	1537427	N13 / B0	*ebpS*	gTc/gCc	Asp—Ala	Elastin-binding protein	Cell wall biogenesis, degradation	Bacterial adhesion to endothelial cells [30]
**CASE 2**	1138065	N13 / B0	*polX*	Ctt/Ttt	Leu—Phe	DNA polymerase	Cellular process	
**CASE 2**	1951225	N13 / B0	*epiB*	aTa/aAa	Tyr—Leu	Lantibiotic epidermin biosynthesis protein	Cellular process	Toxin production and resistance, http://aureowiki.med.uni-greifswald.de/SAUSA300_RS09665
**CASE 2**	2201428	N13 / B0	*cshA*	Acc/Gcc	Gly—Ala	DEAD/H box family ATP-dependent RNA helicase	DNA metabolism	Inactivation results in dysregulation of biofilm formation and hemolysis through modulation of *agr* mRNA stability [31]
**CASE 2**	607085	N13 / B0	SAUSA300_RS02885	gGt/gTt	Gly—Val	Haloacid dehalogenase-like family (GO)	Energy metabolism	Virulence and adhesion [32]
**CASE 2**	1441189	N13 / B0	*arlS*	aTg/aAg	His—Lys	Two-component sensor histidine kinase	Signal transduction	Regulates virulence proteins [33]
**CASE 2**	385424	N13 / B0	SAUSA300_RS01770	cCa/cGa	Trp—Arg	BglG family transcriptional antiterminator	Transport and binding	Up-regulated in *rsp* mutant [34]
**CASE 2**	2486418	N13 / B0	*lctP*	aGt/aTt	Thr—Ile	L-lactate permease	Transport and binding	Adaption to fluctuating oxygen environments [35]
**CASE 2**	1794287	N13 / B0	SAUSA300_RS08930	aaG/aaA	Leu—Lys	Membrane protein	Unknown function	
**CASE 3**	302945	N108, N85, N45, N2 / B0	SAUSA300_RS01340	Gtt/Ttt	Val—Phe	TagB2-CDP-ribitol ribophosphatase	Hypothetical protein	Teichoic acid biosynthesis protein
**CASE 4**	1578750	N56 / N0, B0	SAUSA300_RS07715	Aaa/Gaa	Phe—Glu	phiSLT ORF 78B-like protein	Regulatory functions	Proteasome regulatory subunit C-terminal
**CASE 6**	357524	N25 / N0, B0	SAUSA300_RS01635	cCa/cGa	Pro—Arg	5'-nucleotidase	Biosynthesis of cofactors, prosthetic groups, carriers	Involved in exoproteome virulence which is modulated by Sae/aureolysin [36]
**CASE 7**	409155	N104 / B0	*metE*	gAa/gGa	Phe—Gly	Homocysteine methyltransferase	Amino acid metabolism	
**CASE 7**	1893514	N104 / B0	*pldB*	aGc/aTc	Ala—Ile	Lysophospholipase	Biosynthesis of cofactors, prosthetic groups, and carriers	Regulated by SgrS which regulates carbon metabolism and virulence [37]
**CASE 7**	2149003	N104 / B0	*agrA*	gAa/gGa	Glu—Gly	DNA-binding response regulator	Central intermediary metabolism	Regulates expression of several virulence genes [38], mutation in this gene during *S*. *aureus* soft skin infection [10]
**CASE 7**	2612136	N104 / B0	SAUSA300_RS13450	tCc/tTc	Gly—Phe	Lipase_3	Hypothetical protein	Lipases enable *S*. *aureus* to invade and destroy host tissues [39]
**CASE 7**	2014786	N104 / B0	*tagH*	tGc/tAc	Ala—Tyr	Teichoic acid ABC transporter	Transport and binding	Cell wall component involved in virulence [40]
**CASE 8**	2775333	N25 / N0, B0	*clfB*	ATCCGGG/TTCTGGA	Asp-Phe, Pro—Trp, Asp—Ile	Clumping factor B	Cell envelope	Promotes bacterial adherence hemodialysis tubing, contributing to the pathogenicity of biomaterial-related infections [41]
**CASE 8**	2775381	N25 / N0, B0	*clfB*	gTC/aTC	Asp—Ile	Clumping factor B	Cell envelope	

**Table 5:** Overview of SNP analysis.

	CASE 1	CASE 2	CASE 3	CASE 4 N56, B0	CASE 4 N326, N208	CASE 5	CASE 6^A^	CASE 7 N104, B0	CASE 8
**TIMEPOINTS**	4	2	5	3	3	2	4	3	3
**DAY FARTHEST FROM BACTEREMIA**	15	13	112	56	118	1	122	104	25
**ISOLATES**	8	4	9	7	3	4	7	7	7
**SYNONYMOUS**	2		1	1	NA				
**NON-SYNONYMOUS**	2	10	1		NA	5	1	6	3
**STOP LOST**					NA				
**STOP GAINED**					NA				
**NON-CODING**	3	3	1		NA		4	2	
**PLASMID SYNONYMOUS**		4			NA	1			
**PLASMID NON-SYNONYMOUS**		3			NA	1			
**PLASMID NON-CODING**		1			NA				
**TOTAL SNPS**	7	21	3	1	9	7	5	8	2
**MEAN PAIRWISE DISTANCE (SNP)**	7	22	4	0	4	0	3 (89)	11	1
**RATE OF DIVERSITFICATION (SNPS/YEAR)**	170	590	10	7	28	2555	15	28	29

^A^ SNP analysis completed using only N25, N0, B0.

mean pairwise distance SNP shown in () calculated using all four isolates (including N122).

### Subject-specific details of the genomic analyses

#### Case 1

Seven SNPs differed among the four time points. All four isolates had one mutation in the membrane protein SAUSA300_RS01130, relative to the USA300 reference sequence, changing the start codon to a tyrosine and presumably resulting in loss of translation of this protein.

#### Case 2

Thirteen SNPs were identified between the N13 and B0 isolates on the genomic backbone and 8 mutations were found on the plasmid pUSA01 (see S6 Table). Multiple TIGRFAM protein categories were affected including transport and binding (SAUSA300_RS01770 and lctP) and signal transduction (*arlS*).

#### Case 3

Three SNPs differed among the five isolates. The intergenic SNP was located ~30 bp upstream of the histidine tRNA ligase, which could result in change of transcription of this gene.

#### Case 4

Over the 326-day timespan the subject had an additional MRSA bacteremia episode at 201 days prior to the B0 event. The bloodstream isolate from this infection was not available. The sequencing results for this subject showed that strains N326/N229/N208 were more similar to one another (~6 SNPs detected) than to the other three isolates (N56/N0/B0), with ~1000 SNPs separating the two sets. Although nasal swabs were collected on days -184 and -142, *S*. *aureus* could not be isolated from either. Two of the three strains sequenced from the N56 time point were MLST 87 and one strain was MLST 5. In contrast, at the N0 time point, all three of the strains sequenced were MLST 5. Only one SNP differed among the N56/N0/B0 strains.

#### Case 5

No mutations were identified between the single nasal and bloodstream isolates (N0 and B0). Although an N13 swab was MRSA positive by Cepheid PCR in the clinical lab, no *S*. *aureus* isolates could subsequently be isolated from the heavy mixed growth from the paired, frozen swab.

#### Case 6

This subject had persistently MRSA-colonized nares documented by the clinical laboratory for 17 months prior to the first sequenced isolate. The N122 isolate’s mean pair-wise distance was ~220 SNPs compared to the other three time points (N25, N0, B0), and therefore not included in SNP comparison. The single non-synonymous mutation resulted in an amino acid substitution in a 5’-nucleotidase.

#### Case 7

Three time points were included in the genomic data analysis (N104/N1/B0). Whereas the N104 and B0 samples clustered together (Fig 2), the three N1 isolates did not cluster with any other isolates from Case 7 and were instead categorized as MLST 87. The Case 4 MLST87 isolates and the Case 7 MLST87 isolates differed by 209–236 SNPs. The N1 isolates were excluded from SNP analysis. The N104 and B0 isolates differed by 8 SNPs, including one in the *agrA* gene, a master-regulator of *S*. *aureus* virulence [42–44]. Because RNAIII expression levels are commonly used to assess *agrA* function, we quantified RNAIII and *gyrB* (housekeeping gene) mRNA by qRT-PCR assay [13] of *in vitro* cultures of N104, B0, USA100, and USA300. Both the N104 and B0 isolates had relatively low levels of expression compared to either the USA300 or USA100 strains, however, the B0 expression levels were reduced 5-fold compared to N104 (p = 0.04; Table 6).

**Table 6:** qRT-PCR results for *agr* expression in Case 7.

	2^(gyrB Ct–RNAIII Ct)^	Lower bound	Upper bound	p-value relative to Case 7 –N104
USA300	68.7	26.0	123.0	0.0002
USA100	24.7	5.9	91.5	0.0001
VA104-N104	0.011	0.003	0.026	NA
VA104-B0	0.0018	0.0007	0.0042	0.038

#### Case 8

Nasal isolates from two time points and one blood isolate were sequenced (N25/N0/B0). Although two earlier nasal swabs were positive for MRSA by Cepheid PCR (N58 and N80), no *S*. *aureus* colonies were recovered from either swab. Three codons, all non-synonymous, differed among the isolates. Two mutations were in the gene *clfB* (clumping factor B, TIGRFAM- cell envelope), which plays a role in nares colonization [45]. The third mutation was located in a hypothetical phage membrane protein located near to lukS-PV on the genome (TIGRFAM—DNA metabolism).

## Discussion

This study provides longitudinal genomic data comparing nasal colonizing *S*. *aureus* strains collected over 1–326 days prior to the onset of bloodstream infection with the subsequent bloodstream isolates in eight patients. We report several key findings. First, colonization progressed to bloodstream infection without acquisition of new virulence genes in all eight cases. Second, the ratio of non-synonymous to synonymous SNPs was 5.5, consistent with selective pressure on the organisms to evolve in the nasal niche. Third, the most common genes that acquired SNPs were those coding for membrane transport functions, although a larger study is needed to determine whether SNPs are statistically more likely to accumulate in particular categories of genes. Fourth, in one case, mutation in the *agr* gene, with associated abrogation of function, was associated with progression to bacteremia. Finally, when triplicate nasal isolates from a single time point were compared to one another, 2.1 +/- 6.5 SNPs were observed, compared with 26 +/- 66 SNPs observed between time points (within one subject), indicating that most of the observed sequence variation between time points was due to strain evolution, rather than selection of variable strains from within the population.

The results of our genomic analyses show that the *S*. *aureus* strains colonizing the nares were closely related to blood isolates from the same individual, confirming results of previous studies [5, 9, 46]. Isolates collected from a given subject were more similar to each other (<26 SNPs across time points within each individual) than to isolates collected from other subjects (>1000 SNPs between MLST 5 subjects and >650 SNPs between MLST 8 subjects). Progression from colonization to bacteremia occurred without acquisition of new virulence genes, similar to the study by Calderwood, Desjardins [47]. However, in our study nonsynonymous SNPs were identified in two regulators of virulence proteins, *argA* (Case 7) and *arlS* (Case 2), as well as other proteins associated with virulence (*epiB*, *tagH*, and *SAUSA300_RS01635*). For these cases, it is possible that alterations in virulence gene expression resulted in strains with greater propensity to cause bacteremia [33, 38, 42].

Our work represents the largest longitudinal study of the genomics of *S*. *aureus* colonization prior to invasive infection of humans. Similar to Young, et al., and Azarian, et al. [9, 10] we found a high ratio of non-synonymous to synonymous SNPs, indicating that genomic evolution from carriage to bacteremia involves some selective pressure. We showed evolution of virulence genes, and a mutation on the *agr* regulatory gene, which abrogated its function.

Several studies [48, 49] have suggested the possibility that more than one *S*. *aureus* strain might simultaneously colonize the nasal cavities. Indeed, we found evidence of colonization by multiple strains in Case 4, a persistently MRSA colonized subject who experienced two bacteremia episodes separated by ~200 days. Our results suggest that treatment of the first episode with vancomycin may have temporarily suppressed *S*. *aureus* colonization of the nares. The nasal carriage strain from the first bacteremia episode differed from the carriage/bacteremia isolates from the second bacteremia episode by ~1000 SNPs (Fig 2). This suggests replacement of the first strain by a different strain, as Azarian observed in two of the four patients in their cohort with MRSA recurrent soft tissue infections [10]. At the N56 time point MLST 87 and MLST 5 were present in the nares, but the MLST 5 strain replaced the MLST 87 strain to become the sole nasal strain and bloodstream isolate at Day 0. Similarly, in Case 7 the three isolates sequenced at N104 matched the blood isolate strain (~10 SNPs different comparing N104 to B0), however, the three nasal isolates at N1 were all identified as MLST 87. In both cases, it is likely that MLST 87 was out-competed by an MLST 5 strain. There was no epidemiologic evidence of transmission of MLST 87 between the two subjects. MLST 87 is an infrequently isolated strain of MRSA [50].

In all cases, the profiles of virulence and antibiotic resistance genes inferred by *in silico* analysis remained constant throughout the study and, within an individual, did not differ between bacteremia and nasal-carriage isolates. In agreement with Varshney et al., the virulence gene profiles of the USA300 (MLST 8) strains contained fewer staphylococcal enterotoxin genes than did the USA100 (MLST 5) strains [51].

Although the isolates identified from within each subject were relatively similar, SNPs between the nasal isolates and bacteremia isolate were identified in 7 of 8 cases. Similar modes of evolution (SNPs in membrane transport genes) were noted compared to studies that exclusively investigated MLST 8 strains [10]. We identified several notable mutations. For example, mutations in *arlS* and *agrA*, which regulate transcription of several virulence factor genes, were identified in Case 2 and Case 7 respectively. One of the intergenic SNPs (Case 6) was located ~20 bp upstream of the alpha-hemolysin transcriptional start site, which could alter transcription and/or translation of the gene. Alterations in alpha-hemolysin expression have been reported to affect virulence in *S*. *aureus* [52]. Additionally, several mutations were identified in genes involved in cell adhesion to the nasal epithelium including *ebpS* (Case 2), *SAUSA300_RS02885* (Case 2), *SAUSA3000_RS01340* (Case 3), and *clfB* (Case 8). Disrupted function of these genes could promote bacteremia because reduced adherence to epithelial cells has been linked to greater likelihood of *S*. *aureus* entering the bloodstream [53]. Overall, these results reveal the wide genotypic landscape that strains can exploit to evolve diverse virulence mechanisms.

Our study has limitations. Although it is the largest study to date of evolution of *S aureus* in the human host, eight subjects remain a relatively small sample. The cases had a high burden of medical morbidity, and may not reflect the evolution of *S*. *aureus* in otherwise healthy hosts. The time between nasal isolates and bloodstream isolates was variable, depending on clinical events for each case. In Case 7, the bloodstream isolate was compared to a nasal isolate from 104 days earlier. It is unknown at what time the decrease in *agr* function that was observed in the bloodstream isolate occurred.

Use of the USA 300 genome as the reference for USA 100 strains may have limited our ability to identify relevant mutations. However, we found that the USA100 strains aligned well with the USA 300 reference. There are no well characterized, reference strains of USA100 with published sequences for reference. Results of additional analyses comparing the isolates to the USA 100 that was sequenced in our laboratory, and to two full length reference sequences, NC002953 MSSA (MSSA 476), and NC002951 COL (Staphylococcus aureus subsp. aureus COL) were similar to the analysis using the USA300 strain.

The successful recovery of *S*. *aureus* from frozen nasal swabs was somewhat surprising. The stress of freezing may have changed the population of *S*. *aureus* that was recoverable from the swab.

In summary, the results of this study show that 1) genomic mutations accumulate at different rates in human nasal carriage strains of *S*. *aureus*, 2) within each patient, less variability in SNP counts was observed between isolates sampled at a single time-point, compared to between time-points, suggesting that selective pressure within the nasal cavities maintains an evolving, but relatively homogenous *S*. *aureus* population genetic structure, 3) co-colonization by different *S*. *aureus* strains and strain replacement occur occasionally, 4) in a relatively healthy host, deleterious mutation of the *agrA* gene was associated with progression to bacteremia, and 5) progression of *S*. *aureus* colonization to nosocomial infection in patients with extensive co-morbidity is seldom due to acquisition of new virulence determinants, such as antibiotic resistance cassettes or toxin-encoding genes.

## Supporting information

S1 Table *De novo* genome assembly statistics. (DOCX)

S2 Table Pairwise comparison of SNPs between Cases using the blood isolates (B0). (DOCX)

S3 Table Antibiotic resistance genes and phenotypic resistance profiles. (DOCX)

S4 Table Accumulated SNPs chromosome (intergenic regions). (DOCX)

S5 Table Accumulated Synonymous SNPs. (DOCX)

S6 Table Accumulated SNPs on plasmid pUSA01. (DOCX)

S7 Table GenBank accession numbers for each isolate. (DOCX)

S1 Fig Phylogenetic relationships between S. aureus nasal and blood isolates. Whole-genome multiple sequence alignments were generated and a maximum-likelihood phylogenetic tree calculated using Fasttree, as described in the text. This cladogram depicts the predicted branching patterns and bootstrap support of branches for all genomic sequences included in this study.
(PDF)

## References

[1] Fowler VG Jr, Sanders LL, Sexton DJ, Kong L, Marr KA, Gopal AK, et al Outcome of *Staphylococcus aureus* bacteremia according to compliance with recommendations of infectious diseases specialists: experience with 244 patients. Clin Infect Dis. 1998;27(3):478–86. . 9770144 10.1086/514686

[2] Lahey T, Shah R, Gittzus J, Schwartzman J, Kirkland K. Infectious diseases consultation lowers mortality from *Staphylococcus aureus* bacteremia. Medicine (Baltimore). 2009;88(5):263–7. doi: 10.1097/MD.0b013e3181b8fccb ; PubMed Central PMCID: PMCPMC2881213. 19745684 10.1097/MD.0b013e3181b8fccb PMC2881213

[3] Wyllie DH, Crook DW, Peto TE. Mortality after *Staphylococcus aureus* bacteraemia in two hospitals in Oxfordshire, 1997–2003: cohort study. BMJ. 2006;333(7562):281 doi: 10.1136/bmj.38834.421713.2F ; PubMed Central PMCID: PMCPMC1526943. 16798756 10.1136/bmj.38834.421713.2F PMC1526943

[4] Wertheim HF, Vos MC, Ott A, van Belkum A, Voss A, Kluytmans JA, et al Risk and outcome of nosocomial *Staphylococcus aureus* bacteraemia in nasal carriers versus non-carriers. Lancet. 2004;364(9435):703–5. doi: 10.1016/S0140-6736(04)16897-9 . 15325835 10.1016/S0140-6736(04)16897-9

[5] von Eiff C, Becker K, Machka K, Stammer H, Peters G. Nasal carriage as a source of *Staphylococcus aureus* bacteremia. Study Group. N Engl J Med. 2001;344(1):11–6. doi: 10.1056/NEJM200101043440102 . 11136954 10.1056/NEJM200101043440102

[6] Epstein L, Mu Y, Belflower R, Scott J, Ray S, Dumyati G, et al Risk Factors for Invasive Methicillin-Resistant *Staphylococcus aureus* Infection After Recent Discharge From an Acute-Care Hospitalization, 2011–2013. Clin Infect Dis. 2016;62(1):45–52. doi: 10.1093/cid/civ777 . 26338787 10.1093/cid/civ777 PMC6557163

[7] van Belkum A, Emonts M, Wertheim H, de Jongh C, Nouwen J, Bartels H, et al The role of human innate immune factors in nasal colonization by *Staphylococcus aureus*. Microbes Infect. 2007;9(12–13):1471–7. doi: 10.1016/j.micinf.2007.08.003 . 17913546 10.1016/j.micinf.2007.08.003

[8] Verkaik NJ, de Vogel CP, Boelens HA, Grumann D, Hoogenboezem T, Vink C, et al Anti-staphylococcal humoral immune response in persistent nasal carriers and noncarriers of *Staphylococcus aureus*. J Infect Dis. 2009;199(5):625–32. doi: 10.1086/596743 . 19199541 10.1086/596743

[9] Young BC, Golubchik T, Batty EM, Fung R, Larner-Svensson H, Votintseva AA, et al Evolutionary dynamics of *Staphylococcus aureus* during progression from carriage to disease. Proc Natl Acad Sci U S A. 2012;109(12):4550–5. doi: 10.1073/pnas.1113219109 ; PubMed Central PMCID: PMCPMC3311376. 22393007 10.1073/pnas.1113219109 PMC3311376

[10] Azarian T, Daum RS, Petty LA, Steinbeck JL, Yin Z, Nolan D, et al Intrahost Evolution of Methicillin-Resistant *Staphylococcus aureus* USA300 Among Individuals With Reoccurring Skin and Soft-Tissue Infections. J Infect Dis. 2016;214(6):895–905. doi: 10.1093/infdis/jiw242 ; PubMed Central PMCID: PMCPMC4996148. 27288537 10.1093/infdis/jiw242 PMC4996148

[11] Bessesen MT, Kotter CV, Wagner BD, Adams JC, Kingery S, Benoit JB, et al MRSA colonization and the nasal microbiome in adults at high risk of colonization and infection. J Infect. 2015;71(6):649–57. doi: 10.1016/j.jinf.2015.08.008 . 26335708 10.1016/j.jinf.2015.08.008

[12] Charlson ME, Pompei P, Ales KL, MacKenzie CR. A new method of classifying prognostic comorbidity in longitudinal studies: development and validation. J Chronic Dis. 1987;40(5):373–83. . 3558716 10.1016/0021-9681(87)90171-8

[13] Laabei M, Jamieson WD, Massey RC, Jenkins AT. *Staphylococcus aureus* interaction with phospholipid vesicles—a new method to accurately determine accessory gene regulator (agr) activity. PLoS One. 2014;9(1):e87270 doi: 10.1371/journal.pone.0087270 ; PubMed Central PMCID: PMCPMC3907525. 24498061 10.1371/journal.pone.0087270 PMC3907525

[14] Frank DN, Feazel LM, Bessesen MT, Price CS, Janoff EN, Pace NR. The human nasal microbiota and *Staphylococcus aureus* carriage. PLoS One. 2010;5(5):e10598 doi: 10.1371/journal.pone.0010598 ; PubMed Central PMCID: PMCPMC2871794. 20498722 10.1371/journal.pone.0010598 PMC2871794

[15] Frank DN, Spiegelman GB, Davis W, Wagner E, Lyons E, Pace NR. Culture-independent molecular analysis of microbial constituents of the healthy human outer ear. J Clin Microbiol. 2003;41(1):295–303. doi: 10.1128/JCM.41.1.295-303.2003 ; PubMed Central PMCID: PMCPMC149572. 12517864 10.1128/JCM.41.1.295-303.2003 PMC149572

[16] Langmead B, Salzberg SL. Fast gapped-read alignment with Bowtie 2. Nat Methods. 2012;9(4):357–9. doi: 10.1038/nmeth.1923 ; PubMed Central PMCID: PMCPMC3322381. 22388286 10.1038/nmeth.1923 PMC3322381

[17] Diep BA, Gill SR, Chang RF, Phan TH, Chen JH, Davidson MG, et al Complete genome sequence of USA300, an epidemic clone of community-acquired meticillin-resistant *Staphylococcus aureus*. Lancet. 2006;367(9512):731–9. doi: 10.1016/S0140-6736(06)68231-7 . 16517273 10.1016/S0140-6736(06)68231-7

[18] Garrison E, Marth G. Haplotype-based variant detection from short-read sequencing. Preprint at arXiv:12073907v2 [q-bioGN]. 2012.

[19] Cingolani P, Patel VM, Coon M, Nguyen T, Land SJ, Ruden DM, et al Using Drosophila melanogaster as a Model for Genotoxic Chemical Mutational Studies with a New Program, SnpSift. Front Genet. 2012;3:35 doi: 10.3389/fgene.2012.00035 ; PubMed Central PMCID: PMCPMC3304048. 22435069 10.3389/fgene.2012.00035 PMC3304048

[20] Haft DH, Selengut JD, White O. The TIGRFAMs database of protein families. Nucleic Acids Res. 2003;31(1):371–3. ; PubMed Central PMCID: PMCPMC165575. 12520025 10.1093/nar/gkg128 PMC165575

[21] Aureowiki [cited 2017 March 2]. Available from: http://aureowiki.med.uni-greifswald.de/Main_Page.

[22] Tritt A, Eisen JA, Facciotti MT, Darling AE. An integrated pipeline for de novo assembly of microbial genomes. PLoS One. 2012;7(9):e42304 doi: 10.1371/journal.pone.0042304 ; PubMed Central PMCID: PMCPMC3441570. 23028432 10.1371/journal.pone.0042304 PMC3441570

[23] Joensen KG, Scheutz F, Lund O, Hasman H, Kaas RS, Nielsen EM, et al Real-time whole-genome sequencing for routine typing, surveillance, and outbreak detection of verotoxigenic *Escherichia coli*. J Clin Microbiol. 2014;52(5):1501–10. doi: 10.1128/JCM.03617-13 ; PubMed Central PMCID: PMCPMC3993690. 24574290 10.1128/JCM.03617-13 PMC3993690

[24] Enright MC, Day NP, Davies CE, Peacock SJ, Spratt BG. Multilocus sequence typing for characterization of methicillin-resistant and methicillin-susceptible clones of *Staphylococcus aureus*. J Clin Microbiol. 2000;38(3):1008–15. ; PubMed Central PMCID: PMCPMC86325. 10698988 10.1128/jcm.38.3.1008-1015.2000 PMC86325

[25] Bradley P, Gordon NC, Walker TM, Dunn L, Heys S, Huang B, et al Rapid antibiotic-resistance predictions from genome sequence data for *Staphylococcus aureus* and *Mycobacterium tuberculosis*. Nat Commun. 2015;6:10063 doi: 10.1038/ncomms10063 ; PubMed Central PMCID: PMCPMC4703848. 26686880 10.1038/ncomms10063 PMC4703848

[26] Dominguez SR, Anderson LJ, Kotter CV, Littlehorn CA, Arms LE, Dowell E, et al Comparison of Whole-Genome Sequencing and Molecular-Epidemiological Techniques for *Clostridium difficile* Strain Typing. J Pediatric Infect Dis Soc. 2016;5(3):329–32. doi: 10.1093/jpids/piv020 . 26407257 10.1093/jpids/piv020

[27] Price MN, Dehal PS, Arkin AP. FastTree: computing large minimum evolution trees with profiles instead of a distance matrix. Mol Biol Evol. 2009;26(7):1641–50. doi: 10.1093/molbev/msp077 ; PubMed Central PMCID: PMCPMC2693737. 19377059 10.1093/molbev/msp077 PMC2693737

[28] Huson DH, Scornavacca C. Dendroscope 3: an interactive tool for rooted phylogenetic trees and networks. Syst Biol. 2012;61(6):1061–7. Epub 2012/07/12. doi: 10.1093/sysbio/sys062 . 22780991 10.1093/sysbio/sys062

[29] Laabei M, Uhlemann AC, Lowy FD, Austin ED, Yokoyama M, Ouadi K, et al Evolutionary Trade-Offs Underlie the Multi-faceted Virulence of *Staphylococcus aureus*. PLoS Biol. 2015;13(9):e1002229 doi: 10.1371/journal.pbio.1002229 ; PubMed Central PMCID: PMCPMC4558032. 26331877 10.1371/journal.pbio.1002229 PMC4558032

[30] Jenkins A, Diep BA, Mai TT, Vo NH, Warrener P, Suzich J, et al Differential expression and roles of *Staphylococcus aureus* virulence determinants during colonization and disease. MBio. 2015;6(1):e02272–14. doi: 10.1128/mBio.02272-14 ; PubMed Central PMCID: PMCPMC4337569. 25691592 10.1128/mBio.02272-14 PMC4337569

[31] Oun S, Redder P, Didier JP, Francois P, Corvaglia AR, Buttazzoni E, et al The CshA DEAD-box RNA helicase is important for quorum sensing control in *Staphylococcus aureus*. RNA Biol. 2013;10(1):157–65. doi: 10.4161/rna.22899 ; PubMed Central PMCID: PMCPMC3590232. 23229022 10.4161/rna.22899 PMC3590232

[32] Edwards J, Gehret A, O’Handley S. A Phosphoglycolate Phosphatase Virulence Factor from *Staphylococcus aureus*. FASEB J. 2015;29(721).

[33] Fournier B, Klier A, Rapoport G. The two-component system ArlS-ArlR is a regulator of virulence gene expression in *Staphylococcus aureus*. Mol Microbiol. 2001;41(1):247–61. . 11454217 10.1046/j.1365-2958.2001.02515.x

[34] Das S, Lindemann C, Young BC, Muller J, Osterreich B, Ternette N, et al Natural mutations in a *Staphylococcus aureus* virulence regulator attenuate cytotoxicity but permit bacteremia and abscess formation. Proc Natl Acad Sci U S A. 2016;113(22):E3101–10. doi: 10.1073/pnas.1520255113 ; PubMed Central PMCID: PMCPMC4896717. 27185949 10.1073/pnas.1520255113 PMC4896717

[35] Ferreira MT, Manso AS, Gaspar P, Pinho MG, Neves AR. Effect of oxygen on glucose metabolism: utilization of lactate in *Staphylococcus aureus* as revealed by in vivo NMR studies. PLoS One. 2013;8(3):e58277 doi: 10.1371/journal.pone.0058277 ; PubMed Central PMCID: PMCPMC3589339. 23472168 10.1371/journal.pone.0058277 PMC3589339

[36] Cassat JE, Hammer ND, Campbell JP, Benson MA, Perrien DS, Mrak LN, et al A secreted bacterial protease tailors the *Staphylococcus aureus* virulence repertoire to modulate bone remodeling during osteomyelitis. Cell Host Microbe. 2013;13(6):759–72. doi: 10.1016/j.chom.2013.05.003 ; PubMed Central PMCID: PMCPMC3721972. 23768499 10.1016/j.chom.2013.05.003 PMC3721972

[37] Papenfort K, Vanderpool CK. Target activation by regulatory RNAs in bacteria. FEMS Microbiol Rev. 2015;39(3):362–78. doi: 10.1093/femsre/fuv016 ; PubMed Central PMCID: PMCPMC4542691. 25934124 10.1093/femsre/fuv016 PMC4542691

[38] Abdelnour A, Arvidson S, Bremell T, Ryden C, Tarkowski A. The accessory gene regulator (agr) controls *Staphylococcus aureus* virulence in a murine arthritis model. Infect Immun. 1993;61(9):3879–85. ; PubMed Central PMCID: PMCPMC281089. 8359909 10.1128/iai.61.9.3879-3885.1993 PMC281089

[39] Hu C, Xiong N, Zhang Y, Rayner S, Chen S. Functional characterization of lipase in the pathogenesis of *Staphylococcus aureus*. Biochem Biophys Res Commun. 2012;419(4):617–20. doi: 10.1016/j.bbrc.2012.02.057 . 22369949 10.1016/j.bbrc.2012.02.057

[40] Wanner S, Schade J, Keinhorster D, Weller N, George SE, Kull L, et al Wall teichoic acids mediate increased virulence in *Staphylococcus aureus*. Nat Microbiol. 2017;2:16257 doi: 10.1038/nmicrobiol.2016.257 . 28112716 10.1038/nmicrobiol.2016.257

[41] Ni Eidhin D, Perkins S, Francois P, Vaudaux P, Hook M, Foster TJ. Clumping factor B (ClfB), a new surface-located fibrinogen-binding adhesin of *Staphylococcus aureus*. Mol Microbiol. 1998;30(2):245–57. . 9791170 10.1046/j.1365-2958.1998.01050.x

[42] Painter KL, Krishna A, Wigneshweraraj S, Edwards AM. What role does the quorum-sensing accessory gene regulator system play during *Staphylococcus aureus* bacteremia? Trends Microbiol. 2014;22(12):676–85. doi: 10.1016/j.tim.2014.09.002 . 25300477 10.1016/j.tim.2014.09.002

[43] Cheung GY, Wang R, Khan BA, Sturdevant DE, Otto M. Role of the accessory gene regulator agr in community-associated methicillin-resistant *Staphylococcus aureus* pathogenesis. Infect Immun. 2011;79(5):1927–35. doi: 10.1128/IAI.00046-11 ; PubMed Central PMCID: PMCPMC3088142. 21402769 10.1128/IAI.00046-11 PMC3088142

[44] Boles BR, Horswill AR. Agr-mediated dispersal of *Staphylococcus aureus* biofilms. PLoS Pathog. 2008;4(4):e1000052 doi: 10.1371/journal.ppat.1000052 ; PubMed Central PMCID: PMCPMC2329812. 18437240 10.1371/journal.ppat.1000052 PMC2329812

[45] Wertheim HF, Walsh E, Choudhurry R, Melles DC, Boelens HA, Miajlovic H, et al Key role for clumping factor B in *Staphylococcus aureus* nasal colonization of humans. PLoS Med. 2008;5(1):e17 doi: 10.1371/journal.pmed.0050017 ; PubMed Central PMCID: PMCPMC2194749. 18198942 10.1371/journal.pmed.0050017 PMC2194749

[46] Yu VL, Goetz A, Wagener M, Smith PB, Rihs JD, Hanchett J, et al *Staphylococcus aureus* nasal carriage and infection in patients on hemodialysis. Efficacy of antibiotic prophylaxis. N Engl J Med. 1986;315(2):91–6. doi: 10.1056/NEJM198607103150204 . 3523240 10.1056/NEJM198607103150204

[47] Calderwood MS, Desjardins CA, Sakoulas G, Nicol R, Dubois A, Delaney ML, et al Staphylococcal enterotoxin P predicts bacteremia in hospitalized patients colonized with methicillin-resistant *Staphylococcus aureus*. J Infect Dis. 2014;209(4):571–7. doi: 10.1093/infdis/jit501 ; PubMed Central PMCID: PMCPMC3903375. 24041793 10.1093/infdis/jit501 PMC3903375

[48] Albrecht VS, Limbago BM, Moran GJ, Krishnadasan A, Gorwitz RJ, McDougal LK, et al *Staphylococcus aureus* Colonization and Strain Type at Various Body Sites among Patients with a Closed Abscess and Uninfected Controls at U.S. Emergency Departments. J Clin Microbiol. 2015;53(11):3478–84. doi: 10.1128/JCM.01371-15 ; PubMed Central PMCID: PMCPMC4609677. 26292314 10.1128/JCM.01371-15 PMC4609677

[49] Votintseva AA, Miller RR, Fung R, Knox K, Godwin H, Peto TE, et al Multiple-strain colonization in nasal carriers of *Staphylococcus aureus*. J Clin Microbiol. 2014;52(4):1192–200. doi: 10.1128/JCM.03254-13 ; PubMed Central PMCID: PMCPMC3993518. 24501033 10.1128/JCM.03254-13 PMC3993518

[50] Monecke S, Coombs G, Shore AC, Coleman DC, Akpaka P, Borg M, et al A field guide to pandemic, epidemic and sporadic clones of methicillin-resistant *Staphylococcus aureus*. PLoS One. 2011;6(4):e17936 Epub 2011/04/16. doi: 10.1371/journal.pone.0017936 ; PubMed Central PMCID: PMCPMC3071808. 21494333 10.1371/journal.pone.0017936 PMC3071808

[51] Varshney AK, Mediavilla JR, Robiou N, Guh A, Wang X, Gialanella P, et al Diverse enterotoxin gene profiles among clonal complexes of *Staphylococcus aureus* isolates from the Bronx, New York. Appl Environ Microbiol. 2009;75(21):6839–49. doi: 10.1128/AEM.00272-09 ; PubMed Central PMCID: PMCPMC2772442. 19749060 10.1128/AEM.00272-09 PMC2772442

[52] Fowler VG Jr, Sakoulas G, McIntyre LM, Meka VG, Arbeit RD, Cabell CH, et al Persistent bacteremia due to methicillin-resistant *Staphylococcus aureus* infection is associated with agr dysfunction and low-level in vitro resistance to thrombin-induced platelet microbicidal protein. J Infect Dis. 2004;190(6):1140–9. doi: 10.1086/423145 . 15319865 10.1086/423145

[53] Wertheim HF, Melles DC, Vos MC, van Leeuwen W, van Belkum A, Verbrugh HA, et al The role of nasal carriage in *Staphylococcus aureus* infections. Lancet Infect Dis. 2005;5(12):751–62. doi: 10.1016/S1473-3099(05)70295-4 . 16310147 10.1016/S1473-3099(05)70295-4

