# Supplementary material for: Genomic evolution of Staphylococcus aureus isolates colonizing the nares and progressing to bacteremia
Source: PLoS One. 2018 May 3;13(5):e0195860. doi: 10.1371/journal.pone.0195860 (PMC5933776; doi:10.1371/journal.pone.0195860)
Supplement: S1 Table — (DOCX) [file pone.0195860.s001.docx]

**Supporting Table 1.**

| Case | Contigs | Genome Size | Longest Scaffold | N50 | Raw reads |
| --- | --- | --- | --- | --- | --- |
| USA300 | 32 | 2903289 | 895923 | 589502 | 1481910 |
| Case 1 B0 | 65 | 2892405 | 799187 | 360905 | 2840814 |
| Case 1 N0 | 52 | 2878579 | 419207 | 242370 | 1352674 |
| Case 1 N11 | 38 | 2860436 | 826706 | 342506 | 1611842 |
| Case 1 N15 | 43 | 2874901 | 581619 | 309304 | 2818934 |
| Case 2 B0 | 28 | 2883446 | 895583 | 194999 | 2996016 |
| Case 2 N13 | 27 | 2888397 | 895675 | 326844 | 1896936 |
| Case 3 B0 | 21 | 2807738 | 1040550 | 222503 | 1758032 |
| Case 3 N108 | 24 | 2807817 | 498676 | 218871 | 1687446 |
| Case 3 N45 | 21 | 2806406 | 733341 | 218796 | 3250668 |
| Case 3 N2 | 27 | 2807499 | 927480 | 220446 | 1733816 |
| Case 4 B0 | 37 | 2895320 | 442996 | 228895 | 1886230 |
| Case 4 N0 | 44 | 2897710 | 533057 | 212301 | 1418266 |
| Case 4 N0 v2 | 37 | 2894365 | 981920 | 212213 | 1126346 |
| Case 4 N208 | 35 | 2913722 | 607059 | 405521 | 3388142 |
| Case 4 N326 | 27 | 2908118 | 606975 | 458912 | 4119062 |
| Case 4 N56 | 44 | 2899022 | 533057 | 222479 | 2138336 |
| Case 5 B0 | 47 | 2900729 | 386371 | 168602 | 1692258 |
| Case 5 N0 | 36 | 2898303 | 567560 | 190307 | 1911556 |
| Case 6 B0 | 25 | 2880497 | 606919 | 282201 | 2025812 |
| Case 6 N0 | 27 | 2880535 | 606924 | 221739 | 1898446 |
| Case 6 N122 | 55 | 2875128 | 257683 | 107925 | 3664142 |
| Case 6 N25 | 33 | 2876025 | 569191 | 175659 | 1605450 |
| Case 7 B0 | 45 | 2888711 | 324822 | 212597 | 1975796 |
| Case 7 N1 | 142 | 2752356 | 124295 | 41790 | 1761964 |
| Case 7 N104 | 44 | 2888181 | 324039 | 200946 | 2358328 |
| Case 8 B0 | 34 | 2879003 | 618431 | 420804 | 2872196 |
| Case 8 N0 | 32 | 2878243 | 1068294 | 420724 | 2652664 |
