# Supplementary material for: Genomic evolution of Staphylococcus aureus isolates colonizing the nares and progressing to bacteremia
Source: PLoS One. 2018 May 3;13(5):e0195860. doi: 10.1371/journal.pone.0195860 (PMC5933776; doi:10.1371/journal.pone.0195860)
Supplement: S2 Table — (DOCX) [file pone.0195860.s002.docx]

**Supporting Table 2.**

|  | USA300 | Case 1 | CAse 2 | Case 3 | Case 4 N56, B0 | Case 4 N326, N208 | Case 5 | Case 6 | Case 7 N104, B0 | Case 8 | Case 7 N1 |
| --- | --- | --- | --- | --- | --- | --- | --- | --- | --- | --- | --- |
| USA300 | 0 |  |  |  |  |  |  |  |  |  |  |
| Case 1 | 480 | 5 |  |  |  |  |  |  |  |  |  |
| Case 2 | 103 | 526 | 11 |  |  |  |  |  |  |  |  |
| Case 3 | 20779 | 20618 | 20797 | 3 |  |  |  |  |  |  |  |
| Case 4 N56, b0 | 21069 | 20758 | 21088 | 852 | 0 |  |  |  |  |  |  |
| Case 4 N326, N208 | 21036 | 20818 | 21055 | 386 | 588 | 295 |  |  |  |  |  |
| Case 5 | 21192 | 20881 | 21210 | 1419 | 928 | 1460 | 0 |  |  |  |  |
| Case 6 | 20937 | 20767 | 20957 | 146 | 886 | 129 | 1451 | 67 |  |  |  |
| Case 7 N104, B0 | 20938 | 20697 | 20956 | 897 | 676 | 941 | 1231 | 926 | 6 |  |  |
| Case 8 | 21001 | 20690 | 21020 | 1267 | 768 | 1326 | 796 | 1316 | 1069 | 1 |  |
| Case 7 N1 | 41285 | 41120 | 41293 | 40840 | 40824 | 40814 | 41380 | 40830 | 40612 | 41224 | 0 |
