# Supplementary material for: Genomic evolution of Staphylococcus aureus isolates colonizing the nares and progressing to bacteremia
Source: PLoS One. 2018 May 3;13(5):e0195860. doi: 10.1371/journal.pone.0195860 (PMC5933776; doi:10.1371/journal.pone.0195860)
Supplement: S3 Table — (DOCX) [file pone.0195860.s003.docx]

**Supporting Table 3.**

|  | **Case 1** | | **Case 2** | | **Case 3** | | **Case 4** | | **Case 5** | | **Case 6** | | **Case 7** | | **Case 8** | |
| --- | --- | --- | --- | --- | --- | --- | --- | --- | --- | --- | --- | --- | --- | --- | --- | --- |
|  | B0 | P | B0 | P | B0 | P | B0 | P | B0 | P | B0 | P | B0 | P | B0 | P |
| Ciprofloxacin | R | R | S | S | R | R | R | R | S | R | R | R | S | R | R | R |
| Clindamycin | S | NA | S | NA | R | NA | R | NA | R | NA | R | NA | R | NA | R | NA |
| Erythromycin | R | NA | R | NA | R | NA | R | NA | R | NA | R | NA | R | NA | R | NA |
| FusidicAcid | S | NA | S | NA | S | NA | S | NA | S | NA | S | NA | S | NA | S | NA |
| Gentamicin | S | NA | S | NA | S | NA | S | NA | S | NA | S | NA | S | NA | S | NA |
| Methicillin | S | R | R | R | S | S | R | R | R | R | R | R | R | R | R | R |
| Mupirocin | S | NA | S | NA | S | NA | S | NA | S | NA | S | NA | S | NA | S | NA |
| Penicillin | R | NA | R | NA | S | NA | R | NA | R | NA | R | NA | R | NA | R | NA |
| Rifampicin | S | NA | S | NA | S | NA | S | NA | S | NA | S | NA | S | NA | S | NA |
| Tetracycline | S | S | S | S | S | S | S | NA | S | S | S | S | S | S | S | S |
| Trimethoprim | S | S | S | S | S | S | S | S | S | S | S | S | S | S | S | S |
| Vancomycin | S | S | S | S | S | S | S | S | S | S | S | S | S | S | S | S |

Resistance predictions from the genotype are presented for blood isolates (B0). The phenotypic resistance pattern (P), obtained by Vitek testing in the clinical laboratory, is presented for the blood isolates. S=sensitive, R-resistant, NA=data not available.
