# Supplementary material for: Genomic evolution of Staphylococcus aureus isolates colonizing the nares and progressing to bacteremia
Source: PLoS One. 2018 May 3;13(5):e0195860. doi: 10.1371/journal.pone.0195860 (PMC5933776; doi:10.1371/journal.pone.0195860)
Supplement: S4 Table — (DOCX) [file pone.0195860.s004.docx]

**Supporting Table 4.**

| Case | Position | Codon | Gene Name/Function |
| --- | --- | --- | --- |
| Case 1 | 414834 | A/G |  |
| Case 1 | 1590676 | T/C |  |
| Case 1 | 2241398 | C/T |  |
| Case 2 | 14415 | G/T |  |
| Case 2 | 580884 | T/C |  |
| Case 2 | 1696588 | C/T | ~20 bp upstream of elongation factor 4 |
| Case 2 | 1892654 | A/G |  |
| Case 3 | 1739187 | C/A | ~ 30 bp upstream of histidine tRNA ligase |
| Case 6 | 2624743 | C/T |  |
| Case 6 | 2624749 | T/C |  |
| Case 6 | 2624755 | A/T |  |
| Case 6 | 1157652 | C/A | upstream of alpha hemolysin |
| Case 7 | 164917 | G/T |  |
| Case 7 | 1957710 | T/A |  |
