# Supplementary material for: Genomic evolution of Staphylococcus aureus isolates colonizing the nares and progressing to bacteremia
Source: PLoS One. 2018 May 3;13(5):e0195860. doi: 10.1371/journal.pone.0195860 (PMC5933776; doi:10.1371/journal.pone.0195860)
Supplement: S5 Table — (DOCX) [file pone.0195860.s005.docx]

**Supporting Table 5.**

| Case | Position | gene | Codon | Amino Acid Change | Gene Name/Function | TIGRFAM category |
| --- | --- | --- | --- | --- | --- | --- |
| Case 1 | 1396571 | *trpA* | ggA/ggG | Gly - Gly | tryptophan synthase subunit alpha | amino acid metabolism |
| Case 1 | 1857321 | SAUSA300_RS09205 | aaG/aaA | Lys - Lys | membrane protein | Transport and binding |
| Case 1 | 2118505-506 | SAUSA300_RS10755 | aGC-aGT | Ser - Ser | ATPase | cellular process |
| Case 3 | 113214 | SAUSA300_RS00535 | taT/taC | Tyr - Tyr | hypothetical protein | hypothetical protein |
| Case 4 | 1578750 | SAUSA300_RS07715 | Aaa/Gaa | Phe - Glu | hypothetical protein | hypothetical protein |
| Case 7 | 2783207 | SAUSA300_RS14300 | TAAAAAAAC/  TAAAAAAAAC | A inserted | hypothetical protein | hypothetical protein |
