# Supplementary material for: Genomic evolution of Staphylococcus aureus isolates colonizing the nares and progressing to bacteremia
Source: PLoS One. 2018 May 3;13(5):e0195860. doi: 10.1371/journal.pone.0195860 (PMC5933776; doi:10.1371/journal.pone.0195860)
Supplement: S6 Table — (DOCX) [file pone.0195860.s006.docx]

**Supporting Table 6**.

| **Case** | **Position pUSA01** | **gene** | **Codon** | **Amino Acid Change** | **Gene Name/Function** | **TIGRFAM category** |
| --- | --- | --- | --- | --- | --- | --- |
| Case 2 | 744 | SAUSA300_RS14705 | gaT/gaC | Asp -Asp | replication protein | unknown |
| Case 2 | 759 | SAUSA300_RS14705 | TgaT/CgaC | Asp- Asp, Asp- Asp | replication protein | unknown |
| Case 2 | 849 | SAUSA300_RS14705 | aaA/aaG | Lys - Lys | replication protein | unknown |
| Case 2 | 875 | SAUSA300_RS14705 | gTc/gCc | Val - Ala | replication protein | unknown |
| Case 2 | 898 | SAUSA300_RS14705 | Gca/Aca | Ala - Thr | replication protein | unknown |
| Case 2 | 918 | SAUSA300_RS14705 | acA/acG | Thr - Thr | replication protein | unknown |
| Case 2 | 930 | SAUSA300_RS14705 | AGA/AA | deletion | replication protein | unknown |
| Case 2 | 2073 | intergenic region | G/A | intergenic | intergenic region |  |
