# Supplementary material for: Genomic evolution of Staphylococcus aureus isolates colonizing the nares and progressing to bacteremia
Source: PLoS One. 2018 May 3;13(5):e0195860. doi: 10.1371/journal.pone.0195860 (PMC5933776; doi:10.1371/journal.pone.0195860)
Supplement: S7 Table — (DOCX) [file pone.0195860.s007.docx]

**Supporting Table 7.**

| GeneBank Accession Number | Contigs accession number | Case_Timepoint |
| --- | --- | --- |
| PHUU00000000.1 | PHUU01000001-PHUU01000033 | BAA-1699 |
| PHUV00000000.1 | PHUV01000001-PHUV01000039 | Case8_N25c |
| PHUW00000000.1 | PHUW01000001-PHUW01000043 | Case8_N25b |
| PHUX00000000.1 | PHUX01000001-PHUX01000040 | Case8_N25a |
| PHUY00000000.1 | PHUY01000001-PHUY01000037 | Case8_N0c |
| PHUZ00000000.1 | PHUZ01000001-PHUZ01000040 | Case8_N0b |
| PHVA00000000.1 | PHVA01000001-PHVA01000032 | Case8_N0a |
| PHVB00000000.1 | PHVB01000001-PHVB01000034 | Case8_B0 |
| PHVC00000000.1 | PHVC01000001-PHVC01000055 | Case7_N1c |
| PHVD00000000.1 | PHVD01000001-PHVD01000045 | Case7_N1b |
| PHVE00000000.1 | PHVE01000001-PHVE01000142 | Case7_N1a |
| PHVF00000000.1 | PHVF01000001-PHVF01000052 | Case7_N104c |
| PHVG00000000.1 | PHVG01000001-PHVG01000050 | Case7_N104b |
| PHVH00000000.1 | PHVH01000001-PHVH01000044 | Case7_N104a |
| PHVI00000000.1 | PHVI01000001-PHVI01000045 | Case7_B0 |
| PHVJ00000000.1 | PHVJ01000001-PHVJ01000030 | Case6_N25 |
| PHVK00000000.1 | PHVK01000001-PHVK01000039 | Case6_N122b |
| PHVL00000000.1 | PHVL01000001-PHVL01000055 | Case6_N122a |
| PHVM00000000.1 | PHVM01000001-PHVM01000034 | Case6_N0c |
| PHVN00000000.1 | PHVN01000001-PHVN01000035 | Case6_N0b |
| PHVO00000000.1 | PHVO01000001-PHVO01000027 | Case6_N0a |
| PHVP00000000.1 | PHVP01000001-PHVP01000025 | Case6_B0 |
| PHVQ00000000.1 | PHVQ01000001-PHVQ01000060 | Case5_N0c |
| PHVR00000000.1 | PHVR01000001-PHVR01000057 | Case5_N0b |
| PHVS00000000.1 | PHVS01000001-PHVS01000036 | Case5_N0a |
| PHVT00000000.1 | PHVT01000001-PHVT01000047 | Case5_B0 |
| PHVU00000000.1 | PHVU01000001-PHVU01000041 | Case4_N56c |
| PHVV00000000.1 | PHVV01000001-PHVV01000046 | Case4_N56b |
| PHVW00000000.1 | PHVW01000001-PHVW01000044 | Case4_N56a |
| PHVX00000000.1 | PHVX01000001-PHVX01000032 | Case4_N326 |
| PHVY00000000.1 | PHVY01000001-PHVY01000037 | Case4_N229 |
| PHVZ00000000.1 | PHVZ01000001-PHVZ01000093 | Case4_N208 |
| PHWA00000000.1 | PHWA01000001-PHWA01000377 | Case4_N0c |
| PHWB00000000.1 | PHWB01000001-PHWB01000037 | Case4_N0b |
| PHWC00000000.1 | PHWC01000001-PHWC01000044 | Case4_N0a |
| PHWD00000000.1 | PHWD01000001-PHWD01000037 | Case4_B0 |
| PHWE00000000.1 | PHWE01000001-PHWE01000024 | Case3_N89 |
| PHWF00000000.1 | PHWF01000001-PHWF01000035 | Case3_N49 |
| PHWG00000000.1 | PHWG01000001-PHWG01000026 | Case3_N2c |
| PHWH00000000.1 | PHWH01000001-PHWH01000072 | Case3_N2b |
| PHWI00000000.1 | PHWI01000001-PHWI01000027 | Case3_N2a |
| PHWJ00000000.1 | PHWJ01000001-PHWJ01000032 | Case3_N108c |
| PHWK00000000.1 | PHWK01000001-PHWK01000045 | Case3_N108b |
| PHWL00000000.1 | PHWL01000001-PHWL01000024 | Case3_N108a |
| PHWM00000000.1 | PHWM01000001-PHWM01000021 | Case3_B0 |
| PHWN00000000.1 | PHWN01000001-PHWN01000085 | Case2_N13c |
| PHWO00000000.1 | PHWO01000001-PHWO01000128 | Case2_N13b |
| PHWP00000000.1 | PHWP01000001-PHWP01000027 | Case2_N13a |
| PHWQ00000000.1 | PHWQ01000001-PHWQ01000028 | Case2_B0 |
| PHWR00000000.1 | PHWR01000001-PHWR01000102 | Case1_N15c |
| PHWS00000000.1 | PHWS01000001-PHWS01000082 | Case1_N15b |
| PHWT00000000.1 | PHWT01000001-PHWT01000039 | Case1_N15a |
| PHWU00000000.1 | PHWU01000001-PHWU01000038 | Case1_N11 |
| PHWV00000000.1 | PHWV01000001-PHWV01000072 | Case1_N0c |
| PHWW00000000.1 | PHWW01000001-PHWW01000072 | Case1_N0b |
| PHWX00000000.1 | PHWX01000001-PHWX01000052 | Case1_N0a |
| PHWY00000000.1 | PHWY01000001-PHWY01001288 | Case1_B0 |
