# Supplementary figures and images for: Genomic evolution of Staphylococcus aureus isolates colonizing the nares and progressing to bacteremia
Source: PLoS One. 2018 May 3;13(5):e0195860. doi: 10.1371/journal.pone.0195860 (PMC5933776; doi:10.1371/journal.pone.0195860)

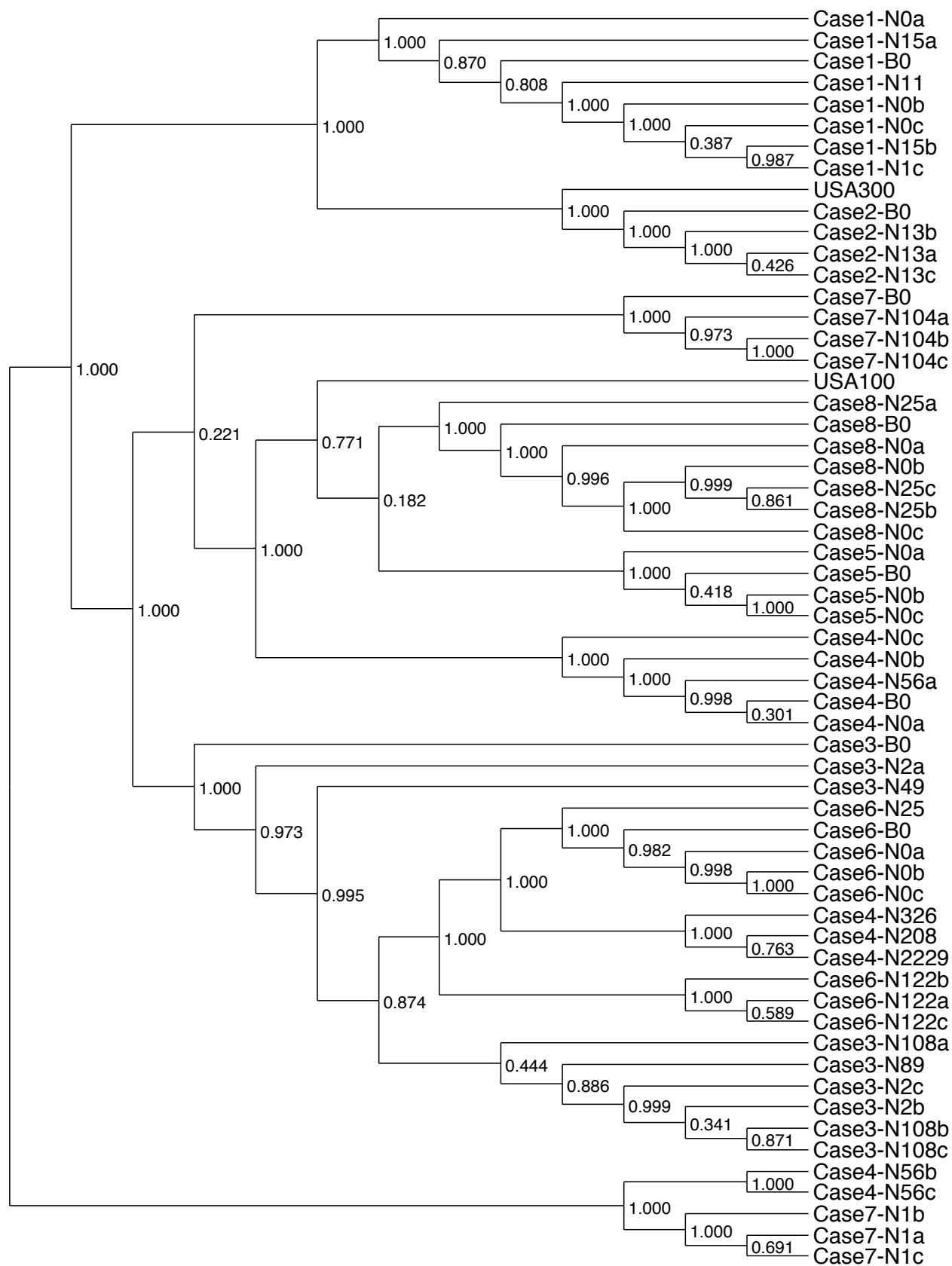

Supplement: S1 Fig — Whole-genome multiple sequence alignments were generated and a maximum-likelihood phylogenetic tree calculated using Fasttree, as described in the text. This cladogram depicts the predicted branching patterns and bootstrap support of branches for all genomic sequences included in this study. (PDF) [file pone.0195860.s008.pdf]
